# Tauroursodeoxycholic acid (TUDCA) improves intestinal barrier function associated with TGR5-MLCK pathway and the alteration of serum metabolites and gut bacteria in weaned piglets

**DOI:** 10.1186/s40104-022-00713-3

**Published:** 2022-06-08

**Authors:** Min Song, Fenglin Zhang, Yiming Fu, Xin Yi, Shengchun Feng, Zhichang Liu, Dun Deng, Qiang Yang, Miao Yu, Canjun Zhu, Xiaotong Zhu, Lina Wang, Ping Gao, Gang Shu, Xianyong Ma, Qingyan Jiang, Songbo Wang

**Affiliations:** 1 Guangdong Provincial Key Laboratory of Animal Nutrition Control, ALLTECH-SCAU Animal Nutrition Control Research Alliance, and National Engineering Research Center for Breeding Swine Industry, College of Animal Science, South China Agricultural University, Guangzhou, 510642 P. R. China; 2 Institute of Animal Science, Guangdong Academy of Agricultural Sciences, State Key Laboratory of Livestock and Poultry Breeding, Key Laboratory of Animal Nutrition and Feed Science in South China, Ministry of Agriculture and Rural Affairs, Guangdong Key Laboratory of Animal Breeding and Nutrition, Guangdong Engineering Technology Research Center of Animal Meat Quality and Safety Control and Evaluation, Guangzhou, 510640 P. R. China; 3 Maoming Branch, Guangdong Laboratory for Lingnan Modern Agricultural Science and Technology, Maoming, 525000 P. R. China

**Keywords:** Gut bacteria, Intestinal barrier function, Serum metabolites, Tauroursodeoxycholic acid (TUDCA), TGR5-MLCK pathway, Weaned piglets

## Abstract

**Background:**

Tauroursodeoxycholic acid (TUDCA), a hydrophilic bile acid, is the main medicinal component of bear bile and is commonly used to treat a variety of hepatobiliary diseases. Meanwhile, TUDCA has been shown to modulate the intestinal barrier function and alleviate DSS-induced colitis in mice. However, the effect of TUDCA on the intestinal barrier of weaned piglets remains largely unclear.

**Methods:**

The weaned piglets and porcine IPEC-J2 intestinal epithelial cells were used to investigate the effects of TUDCA on intestinal barrier function in weaned piglets and explore the possible underlying mechanisms. In vivo*,* 72 healthy weaned piglets were randomly allocated into 2 groups according to their gender and body weight, and piglets were fed the basal diet with 0 (control, CON) and 200 mg/kg TUDCA for 30 d, respectively. Three female and three male piglets reflecting the average bodyweight were slaughtered in each group and samples were collected. In vitro, IPEC-J2 cells were subjected to 100 μmol/L TUDCA to explore the possible underlying mechanisms.

**Results:**

Our results demonstrated that dietary TUDCA supplementation significantly reduced the diarrhea incidence of weaned piglets, possibly attributing to the TUDCA-enhanced intestinal barrier function and immunity. In addition, TUDCA supplementation altered serum metabolites and the relative abundance of certain gut bacteria, which might contribute to the improved intestinal barrier function. Furthermore, the in-vitro results showed that TUDCA improved the *E. coli*-induced epithelial barrier impairment of IPEC-J2 cells and increased Takeda G-coupled protein receptor 5 (TGR5) protein expression. However, knockdown of TGR5 and inhibition of myosin light chain kinase (MLCK) pathway abolished the TUDCA-improved epithelial barrier impairment in *E. coli*-treated IPEC-J2 cells, indicating the involvement of TGR5-MLCK in this process.

**Conclusions:**

These findings showed that TUDCA improved intestinal barrier function associated with TGR5-MLCK pathway and the alteration of serum metabolites and gut bacteria in weaned piglets, suggesting the potential application of TUDCA in improving gut health in piglet production.

## Introduction

In the modern pig breeding industry, weaning is the key stage of pig breeding process. Sudden changes in diet and living environment during weaning often cause weaning stress of piglets, which leads to atrophy of intestinal villi, crypt hyperplasia, reduction of gastrointestinal volume and appetite [1, 2]. In addition, the gastrointestinal tract is immature when piglets are weaned, and the secretion of bile and endogenous digestive enzymes such as lipase is insufficient. Thus, lipids in the feed are difficult to be digested and absorbed, leading to diarrhea, decreased immunity, slow growth and even death of weaned piglets.

Bile acids (BAs), derived from cholesterol in the liver, are amphiphilic molecules composed of hydrophilic and hydrophobic groups, and are known for promoting lipid digestion. In addition, more and more evidence has demonstrated that BAs also act as signal molecules to participate in the regulation of bile acid synthesis, glycolipid metabolism, immunity and energy homeostasis by activating bile acid receptors such as farnesoid X receptor (FXR) and Takeda G-coupled protein receptor 5 (TGR5) [3–6].

It has been well characterized that gut microbiota plays a very important role in bile acid metabolism. The bile acids synthesized by the liver are defined as primary bile acids, including chenodeoxycholic acid (CDCA) and cholic acid (CA). The primary bile acids are first conjugated to taurine or glycine, and then they are secreted into the digestive tract after eating [7]. After reaching the ileum, primary bile acids are transformed to secondary bile acids under the action of gut microbiota via deconjugation, dehydrogenation, and dehydroxylation [8, 9]. The secondary bile acids produced include lithocholic acid (LCA), deoxycholic acid (DCA), ursodeoxycholic acid (UDCA), hyodeoxycholic acid (HDCA) and their conjugated forms and isomeride. In addition, accumulating evidence have suggested a crosstalk between the gut microbiota and serum metabolites [7, 9]. Changes in the gut microbiome are often accompanied by alterations in the host’s serum metabolome, and thus have a profound effect on the overall host health [10].

TUDCA, a taurine-conjugated derivative of UDCA, is the main medicinal component of bear bile and is approved by the Food and Drug Administration (FDA) for the treatment of primary biliary cholangitis due to its protective effects on hepatocytes [11]. UDCA is a secondary bile acid, which is produced by gut microbiota through the epimerization of hydroxyl groups of CDCA [12, 13]. The generated UDCA directly enters the liver through the hepato-intestinal circulation, and then conjugated with taurine to form TUDCA [14]. UDCA and TUDCA have similar physiological functions, but TUDCA is more soluble and less toxic than UDCA. Recently, the effects of TUDCA have been shown to extend beyond hepatobiliary disorders. TUDCA is also recognized as a chemical chaperone to enhance protein folding and protect cells from endoplasmic reticulum (ER) stress, and is widely used in clinical and experimental research for the treatment of obesity, liver disease and neurodegenerative diseases [15, 16]. In addition, it has been shown that TUDCA is an agonist of TGR5 and plays an anti-inflammatory role by activating TGR5. Furthermore, it has been reported that TUDCA alleviates the weight loss, colon shortening and increasing inflammatory factors expression in DSS-induced colitis mice [17]. In term of mechanism, TUDCA alleviates mouse colitis by reducing ER stress of intestinal epithelial cells and inhibiting early intestinal epithelial cell apoptosis [18, 19]. However, the effect of TUDCA on the intestinal barrier of weaned piglets has rarely been reported. Based on previous studies and the properties as a bile acid, we hypothesized that: i) TUDCA might improve intestinal barrier function in piglets, thereby improving growth performance and intestinal health of weaned piglets; ii) TUDCA ameliorates *E. coli*-induced impairment of epithelial barrier function in IPEC-J2 cells through TGR5-MLCK signaling pathway. The IPEC-J2, originally derived from neonatal piglet jejunum, is a noncancerous and nontransformed cell line. IPEC-J2 has similar characteristics to the porcine intestinal epithelium cells and is often used as an experimental model of porcine intestinal epithelial cells in vitro.

Therefore, the objective of this study is to investigate the effects of TUDCA on intestinal barrier function using in vivo and in vitro models. An in vivo study was carried out to determine the effects of TUDCA on intestinal epithelial barrier function, serum metabolic profiles and gut microbiota in piglets. In vitro, *E. coli-*induced epithelial barrier impairment model of IPEC-J2 cells was used to identify the role of TGR5-MLCK pathway in TUDCA improving intestinal barrier function. Our findings show that TUDCA improves intestinal barrier function associated with TGR5-MLCK pathway and the alteration of serum metabolites and gut bacteria in weaned piglets.

## Materials and methods

### Animals management and experimental design

The animal protocol for this study was conducted with the permission number of SYXK (Guangdong) 2019–0136, and the animal care procedures for this study were performed in accordance with the guidelines of The Animal Ethics Committee of South China Agricultural University.

Seventy-two crossbred healthy weaned piglets (Duroc × Landrace × Yorkshire) were weaned at 21 ± 2 days of age. After 7-day adaptation, piglets (initial body weight of 7.02 ± 0.08 kg) were raised on a local commercial farm (Guangdong Huahong Farming and Husbandry Group Co., Ltd., Yangjiang, Guangdong Province, China). All weaned piglets had free access to the basal diet during the 7-day adaptation period. The based diet (Table 1) was formulated to meet all nutrient requirements recommended by National Research Council (NRC, 2012) [20]. At 28 ± 2 days of the age, piglets were randomly assigned into two groups according to the gender and body weight. Each group had 4 replications (pens) of 9 piglets per replicate (male:female =4:5). The piglets in the control group (CON) were fed with a basal diet, while the piglets in the TUDCA group were fed with the basal diet supplemented with 200 mg/kg TUDCA (Hangzhou Baoji Biotechnology Co. Ltd., Hangzhou, China. The dosage of TUDCA in piglets diet is based on our previous piglets experimental results [21] and the recommended dosage of similar bile acid products). In addition, the basal diet also included 50 mg/kg of quinocetone and 75 mg/kg of chlortetracycline. The experiment lasted 30 d. The piglets were fed three times daily at approximately 08:00 h, 13:00 h and 18:00 h. All piglets had free access to food and water throughout the experimental period.

**Table 1:** Composition and nutrient levels of the basal diet for weaned piglets

Ingredient	Ratio, %
Corn	61.50
Dehulled soybean meal	20.50
Expanded soybean meal	5.00
50% oil powder	3.00
Lactose	3.00
Glucose	2.50
Fish meal	1.50
Vitamin-mineral premix^a^	1.00
Calcium hydrogen phosphate	1.00
Stone powder	1.00
Total	100.00
Chemical composition	
Digestible energy, MJ/kg	14.56
Crude protein, %	18.50
Ca, %	0.73
Available P, %	0.52
Lys, %	1.3
Cys, %	0.78
Thr, %	0.84
Trp, %	0.25

^a^Premix provided per kilogram of diet: 12,000 IU of Vitamin A, 2400 IU of Vitamin D_3_, 60 IU of Vitamin E, 2.0 mg of Vitamin K_3_, 2.0 mg of Vitamin B_1_, 10 mg of VB_2_, 40 mg of niacin, 12.0 mg of Vitamin B_6_, 0.03 mg of Vitamin B_12_, 20.0 mg of d-pantothenic acid, 2.1 mg of folic acid, 0.30 mg of biotin, 500.0 mg of choline chloride, 25.0 mg of Cu, 150.0 mg of Fe, 30.0 mg of Mn, 150.0 mg of Zn, 0.5 mg of I, 0.3 mg of Co, 0.5 mg of Se and 4.0 mg of ethoxyquin

### Growth performance

Piglets were weighed individually at the beginning and end of the experiment and feed intake of piglets in each pen was recorded daily. Average daily gain (ADG), average daily feed intake (ADFI) and feed/gain ratio (F/G) were calculated for the piglets in each pen. Additionally, during the entire experiment, piglets (from 28 to 57 days of age) were monitored for diarrhea symptoms twice a day at 8:00 h and 18:00 h. The severity of diarrhea was assessed by fecal consistency: solid 0, semi-solid 1, semi-liquid 2, liquid 3. Excreting level 2 or 3 feces for two consecutive days was defined as diarrhea [22]. Diarrhea incidence was calculated according to the followed formula:

$$ \mathrm{Diarrhea}\ \mathrm{in}\mathrm{cidence}\ \left(\%\right)=\left[\mathrm{Total}\ \mathrm{number}\ \mathrm{of}\ \mathrm{piglets}\ \mathrm{with}\ \mathrm{diarrhea}\ \mathrm{in}\ \mathrm{each}\ \mathrm{pen}\times \mathrm{Diarrhea}\ \mathrm{days}/\left(\mathrm{Number}\ \mathrm{of}\ \mathrm{piglets}\mathrm{in}\ \mathrm{each}\ \mathrm{pen}\times \mathrm{Experimentdays}\right)\right]\times 100\% $$

### Sample treatment and collection

On day 30 of the experiment, 3 female and 3 male piglets reflecting the average body weight (BW) were selected for sample collection from each group. Meanwhile, we ensured that at least one piglet was selected for sample collection per replicate. Blood samples were collected after 12-h fasting by jugular venipuncture before euthanasia. After incubation at room temperature for 30 min, the blood was centrifuged at 3000 r/min for 15 min, then the serum was separated and stored at − 20 °C for further analysis. The middle sections of jejunum and the ileum were collected and then divided into 2 segments. One segment was fixed in a 4% neutral-buffered formalin solution for intestinal morphological analysis and the other segment was stored at − 80 °C until the extraction of total RNA and protein. The pancreas samples were also collected, and then stored at − 80 °C prior to mRNA analysis. In addition, fecal samples were collected from the rectum near the anus of the slaughtered piglets and then stored at − 80 °C until 16S rRNA gene sequences.

### Intestinal histomorphology analysis

The fixed intestinal segments were dehydrated and then embedded in low-melting paraffin wax. Cross sections of intestinal segments were cut into 5-μm thick histological sections and stained with haematoxylin and eosin (H&E) as previously described [23]. Histological section were examined using a light microscopy.

At least 10 microscopic fields per sample were randomly selected to measure villus height (V) and crypt depth (C) of the small intestine by using Program Image-pro Plus 6.0 (Media Cybernetics Bethesda, MD, USA), and the V/C ratio was calculated. Goblet cells in jejunum and ileum were identified using periodic acid-schiff (PAS) stain described previously [24].

### Measurement of serum lipopolysaccharide (LPS), diamine oxidase (DAO) and immune indices

Serum LPS, DAO and IgG levels and ileal mucosal secretory immunoglobulin A (sIgA) level were detected by porcine LPS, DAO, IgG and sIgA ELISA kit (Nanjing Jiancheng Bioengineering Institute, Nanjing, China) according to the manufacturer’s instructions. The ileal mucosal contents of IL-1β, NF-κB, IL-6, IL-4 and IL-10 were detected by porcine ELISA kit (Shanghai Enzyme-linked Biotechnology Co., Ltd., Shanghai, China) according to the manufacturer’s instructions.

### Western blot (WB) analysis

Expression of tight junction (TJ) proteins in jejunum was determined by WB analysis which was conducted as previously described [25]. Proteins were detected by primary antibodies: anti-Tubulin antibodies (1:5000, Bioss, China), anti-Occludin (OCC) (1:500, Bioss, China), anti-ZO-1 (1:2000, Santa Cruz, USA) and anti-Claudin-1 (1:500, Bioss, China), and then incubated with the secondary antibodies (1:2000, Bioss, China). Finally, chemiluminescence detection was performed with western ECL reagent and band densities were quantified using a FluorChem M Fluorescent Imaging System (ProteinSimple, Santa Clara, CA, USA). The intensity of bands was analyzed and quantified by Image J Sofware (NIH, Bethesda, MD, USA).

### Serum metabolic profiles

Untargeted metabolic profiling of serum samples by liquid chromatography tandem mass spectrometry (LC-MS/MS) was performed to analyze serum metabolic profiles. Serum sample preparation and LC-MS/MS method were performed according to the method described previously [26]. Use the database of Kyoto Encyclopedia of Genes and Genomes (KEGG) to perform functional annotations on the identified metabolites, and then map the annotated metabolites to the KEGG pathway database.

### DNA extraction and 16S rRNA gene sequencing

The total DNA was extracted and purified from fecal samples using an E.Z.N.A.® stool DNA Kit (Omega) following the manufacturer’s instructions. DNA quality was confirmed by agarose gel electrophoresis. The V3–V4 regions of 16S rRNA genes were amplified by PCR using primers 341F (5′-CTCCTACGGGAGGCAGCAGT-3′) and 806R (5′-GGACTACHVGGGTWTCTAAT-3′). Mix the purified products in equal proportions for sequencing using the Illumina Hiseq 2500 platform. The sequencing protocol was performed according to a previous method [27]. Raw sequencing data were processed with QIIME software package. The 16S rRNA gene sequences were selected to compare the relative abundance of bacterial groups. Operational taxonomic units (OTUs) clustering was performed by Uparse software and delimited at a threshold of 97% sequence similarity using USEARCH v.10. The alpha and beta diversity of microflora was determined. In our present study, the alpha diversity was analyzed using Chao1 index and Shannon’s index, while the beta diversity was analyzed by principal component analysis (PCoA). Bacterial species differences between the TUDCA and the CON groups were compared using metastat analysis.

Finally, the bacterial functions were predicted by PICRUSt based on the obtained data, and the differential functions and metabolic pathways between the TUDCA and the CON groups were obtained. In addition, the Spearman correlation was used to analyze the correlation between intestinal differential OTUs and serum differential metabolites.

### Bacterial preparation

The *E. coli K88* strain, purchasing from the China Veterinary Culture Collection Center, was grown in Luria-Bertani (LB) broth. After incubation at 37 °C with shaking overnight, bacteria were 1:100 diluted in fresh LB and grown for 2 h. Bacteria were harvested by centrifugation at 3000×g for 10 min at 4 °C, washed twice with phosphate buffer solution (PBS) and resuspended in RPMI-1640 medium free of antibiotics. The bacterial concentration (colony-forming units, CFU) was determined by serial dilution method.

### Cell culture conditions and treatments

IPEC-J2 cells were cultured in RPMI-1640 medium supplemented with 10% of fetal bovine serum (FBS), 1% of penicillin (100 U/mL), and streptomycin (100 U/mL) in humidified atmosphere with 5% CO_2_ at 37 °C. The medium was changed every 2 days. TUDCA was dissolved in dimethyl sulfoxide (DMSO) for in vitro experiments.

For experiment, IPEC-J2 cells were inoculated into 6-well plates with a density of 3 × 10^5^ cells/cm^2^. After reaching 70% to 80% confluence, cells were cultured with RPMI-1640 medium supplemented with 100 μmol/L TUDCA (Preliminary results showed that 100 μmol/L TUDCA could significantly increase TGR5 protein expression of IPEC-J2 cells, with no significant effect on IPEC-J2 cell viability. At the same time, 100 μmol/L TUDCA has a significant anti-inflammatory effect on LPS-induced macrophages. Thus, 100 μmol/L TUDCA were finally selected in our experiments) or PBS or 10 μmol/L ML-7 for 12 h. Afterward, cells were stimulated with *E. coli K88* (1 × 10^3^ CFU/well) for 6 h.

To silence *TGR5* gene, siRNAs (small interfering RNAs) targeted against *TGR5* were transfected into IPEC-J2 cells for 6 h according to the manufacturer’s instructions. The empty plasmid vector was used as a negative control (CON). Subsequently, the medium was replaced with complete medium and the cells were treated with TUDCA and *E. coli K88*. The efficiency of gene silencing was determined by WB after the treatments.

### Measurement of lactate dehydrogenase (LDH) in cell culture supernatant

The LDH activity of cell culture supernatant was detected by LDH Assay Kit (Nanjing Jiancheng Bioengineering Institute, Nanjing, China) according to the protocol of kit.

### Statistical analysis

Data were statistically analyzed using Sigmaplot 14.0 (Systat Software, Inc., San Jose, CA) and the statistical significance of differences was evaluated by two-tailed Student’s *t*-test. A confidence level of *P* < 0.05 was considered to be statistically significant. All data were expressed as mean value ± standard error of the mean (SEM). Moreover, Variable Importance in Projection (VIP) ≥ 1, and the fold change ≥2 or fold change ≤0.5 were used as indicators to screen the serum differential metabolites between the CON group and the TUDCA group. Spearman correlation was used to analyze the correlation between intestinal differential OTUs and serum differential metabolites.

## Results

### TUDCA supplementation reduced the diarrhea incidence of weaned piglets

As shown in Table 2, compared with the CON group, TUDCA supplementation had no significant effect on the final BW, ADG, ADFI and F/G of weaned piglets. However, TUDCA significantly reduced the diarrhea incidence of weaned piglets.

**Table 2:** Growth performance and diarrhea incidence of weaned piglets

Items^a^	CON	TUDCA	*P*-value
Initial BW, kg, *n* = 36	7.00 ± 0.12	7.06 ± 0.0.71	0.725
Final BW, kg, *n* = 36	18.61 ± 0.47	19.27 ± 3.26	0.360
ADG, g, *n* = 36	386.38 ± 13.14	418.82 ± 14.00	0.096
ADFI, g, *n* = 4	617.54 ± 39.05	670.77 ± 49.05	0.292
F/G, *n* = 4	1.53 ± 0.02	1.50 ± 0.02	0.163
Diarrhea incidence, %, *n* = 4	13.29 ± 1.03	6.85 ± 2.23**	0.005**

^a^ *BW* Body weight, *ADG* Average daily gain, *ADFI* Average daily feed intake, *F/G* Feed/gain ratio. Data are presented as the mean ± SEM. The *P* values were determined using two-tailed Student’s *t*-test (** *P* < 0.01)

### TUDCA supplementation enhanced intestinal barrier function of weaned piglets

**Fig. 1:**
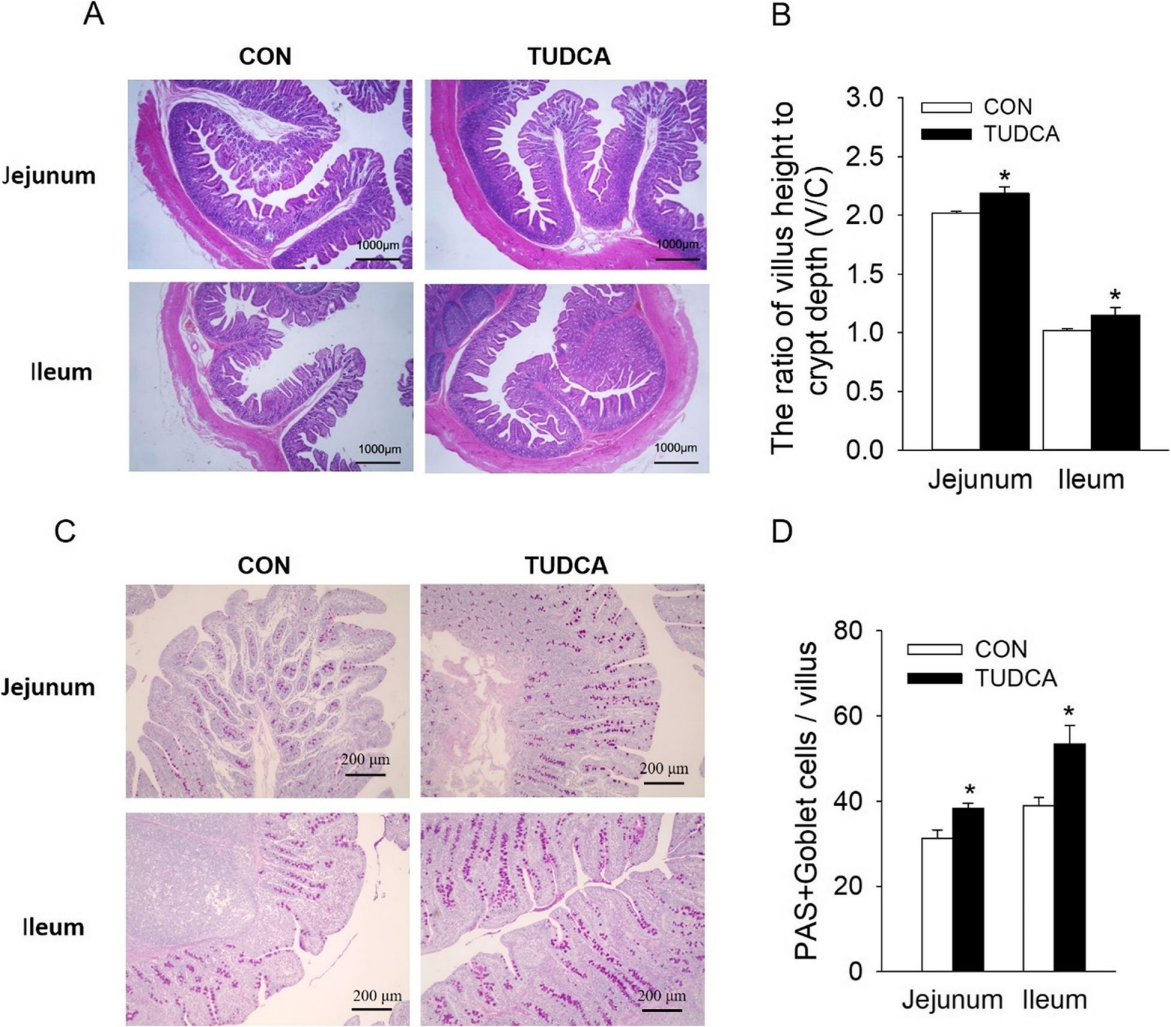
Effects of TUDCA supplementation on the intestinal morphology and mucosa mucopolysaccharide of weaned piglets (*n* = 6). **A** The intestinal morphology of the jejunum and ileum with H&E staining (scale bar, 1000 μm). **B** Statistical diagram of the villus height-to-crypt depth ratio in the jejunum and ileum. **C** The intestinal mucosa mucopolysaccharide of jejunum and ileum stained with periodic acid-schiff (PAS) (scale bar, 200 μm). **D** Statistical diagram of the number of PAS positive cells in each villus. *P < 0.05, versus the CON group

**Fig. 2:**
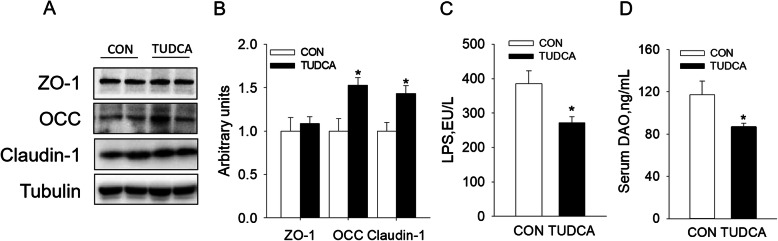
TUDCA supplementation enhanced intestinal barrier function in weaned piglets. **A** and **B** Effects of TUDCA supplementation on expression of tight junction proteins ZO-1, OCC and Claudin-1 in jejunum of weaned piglets. Tubulin was used as the loading control (*n* = 4). **C** and **D** Serum levels of LPS and DAO (*n* = 6). *P < 0.05 versus the CON group

Compared with the CON group, TUDCA supplementation improved the intestinal morphology by increasing the V/C ratios in jejunum and ileum (*P* < 0.05) (Fig. 1A, B). In addition, PAS staining results showed that TUDCA supplementation increased the number of goblet cells secreting mucopolysaccharides in jejunum and ileum (*P* < 0.05, Fig. 1C and D). Furthermore, the expression of TJ proteins, such as OCC and Claudin-1, was higher in jejunum of piglets supplemented with TUDCA (*P* < 0.05, Fig. 2A, B). Accordingly, the intestinal permeability was reduced, which was manifested by the decreased serum LPS and DAO levels (*P* < 0.05, Fig. 2C, D). These above results suggested that dietary supplementation of TUDCA enhanced intestinal barrier function of weaned piglets.

### TUDCA supplementation improved the immunity of weaned piglets

**Fig. 3:**
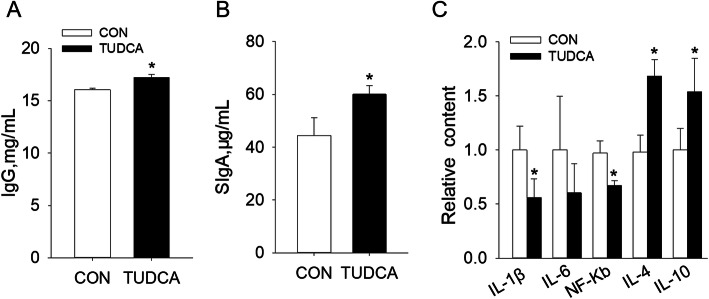
TUDCA supplementation improved the immunity of weaned piglets (*n* = 6). The serum IgG level (**A**) and ileal mucosal sIgA (**B**) were detected by ELISA. **C** The relative content of inflammatory factors IL-1β, IL-6, NF-κb, IL-4 and IL-10. *P < 0.05 versus the CON group

Compared with the CON group, TUDCA supplementation increased the IgG level in serum and the sIgA level in ileum mucosa of weaned piglets (Fig. 3A and B). In addition, TUDCA supplementation reduced the contents of IL-1β and NF-κB and increased the contents of IL-4 and IL-10 in the ileal mucosa of weaned piglets (Fig. 3C). These above results revealed that dietary supplementation of TUDCA reduced intestinal inflammation and improved intestinal immunity of weaned piglets.

### TUDCA supplementation changed the serum metabolic profiles of weaned piglets

**Fig. 4:**
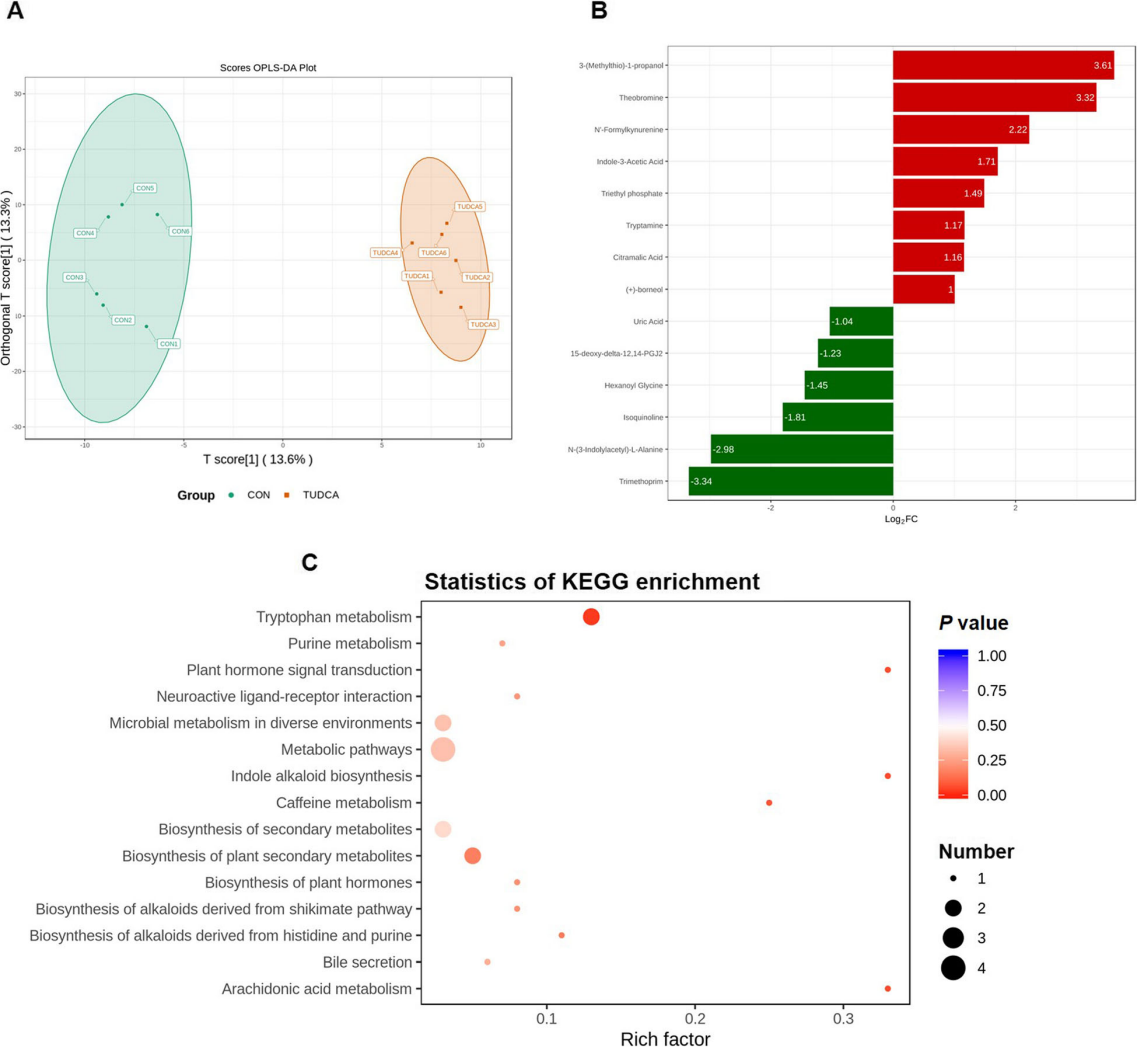
TUDCA supplementation changed serum metabolic profiles of weaned piglets (*n* = 6). **A** The scores plots of serum metabolites of CON group and TUDCA group were obtained by orthogonal partial least squares discriminant analysis (OPLS-DA). **B** The main up-regulated (red-marked) and down-regulated (green-marked) serum differential metabolites. **C** Kyoto Encyclopedia of Genes and Genomes (KEGG) enrichment of serum differential metabolites. The color of the point and the size of the spot represented *P*-value and the number of different metabolites enriched, respectively

In this study, an untargeted UPLC-MS/MS approach was used to comprehensively analyze serum metabolic profiles of weaned piglets. The orthogonal partial least squares-discriminant analysis (OPLS-DA) scores plot showed that there was a clear separation between the CON and TUDCA groups (Fig. 4A), indicating that the serum metabolic profiles of TUDCA group was significantly different from that of CON group. The 14 serum metabolites with the largest change were screened out as shown in Fig. 4B. Among the 14 serum metabolites, the up-regulated serum metabolites included 3-(Methylthio)-1-propanol, theobromine, N′-formylkynurenine, indole-3-acetic acid, triethyl phosphate, tryptamine, citramalic acid and (+)-borneol, and the down-regulated serum metabolites included uric acid, 15-deoxy-δ-12,14-PGJ2, hexanoyl glycine, isoquinoline, N-(3-indolylacetyl)-L-alanine and trimethoprim. In addition, the influences of TUDCA on the metabolic pathways of weaned piglets were explored by using KEGG pathway analysis. As shown in Fig. 4C, TUDCA supplementation altered 15 metabolic pathways, and the predominantly involved metabolic pathways were tryptophan metabolism, microbial metabolism in diverse environments and metabolic pathways.

### TUDCA supplementation altered gut bacteria of weaned piglets at the genus level

**Fig. 5:**
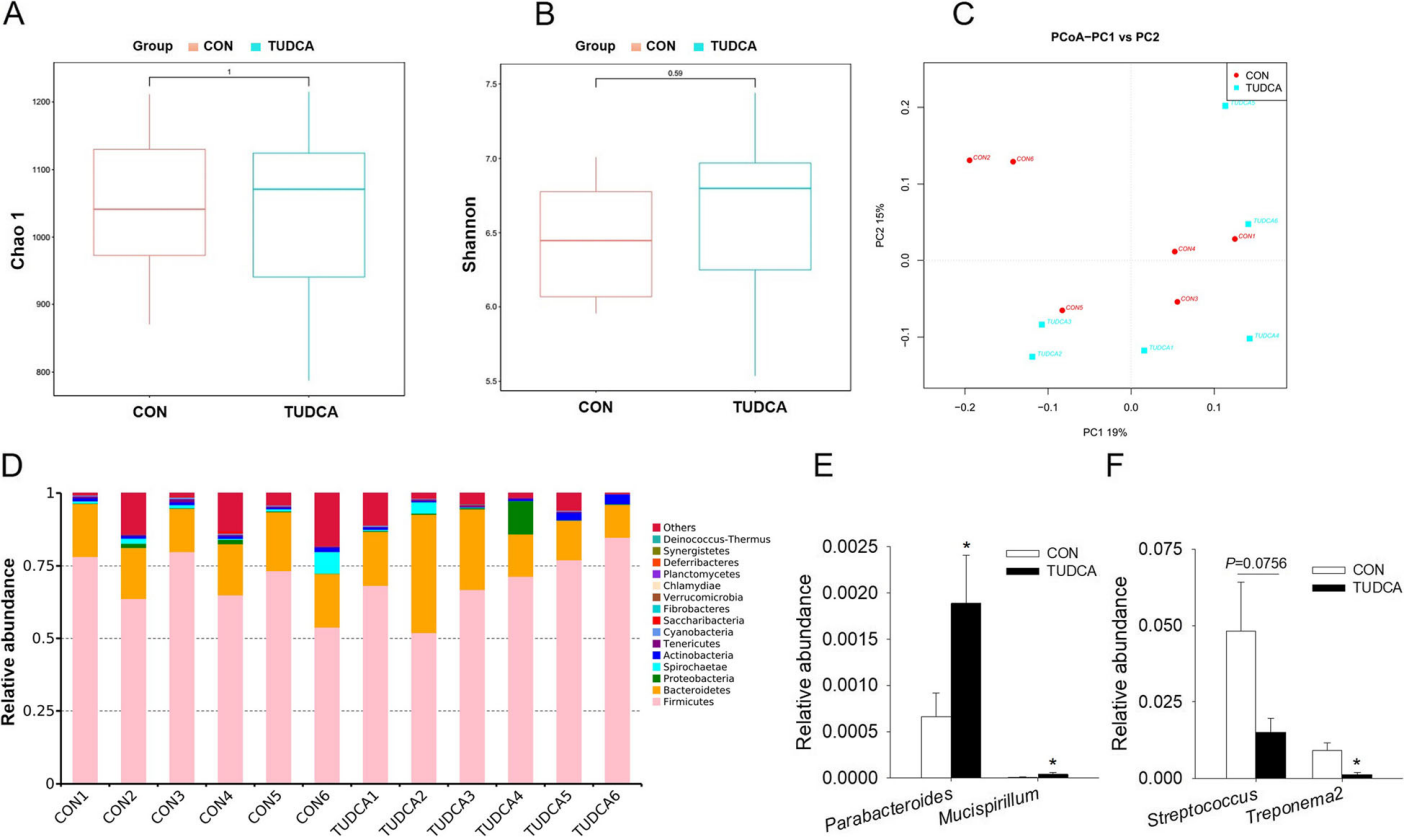
TUDCA supplementation altered gut microbiota of weaned piglets (*n* = 6). **A** and **B** Chao1 value (**A**) and Shannon index (**B**) of gut microbiota, **C** Principal component analysis (PCoA) scores plot for gut microbiota of the CON and TUDCA groups. **D** The relative gut microbiota abundance at the phylum level in the CON and TUDCA groups. **E** and **F** Gut bacteria with significant differences in the genus level after TUDCA supplementation. *P < 0.05 versus the CON group

To investigate the effects of TUDCA supplementation on the gut microbiota of weaned piglets, 16S rRNA gene sequencing was used to evaluate the fecal microbiota composition. As shown in Fig. 5A and B, the parameters of the alpha diversity of gut microbiota, including Chao1 value and Shannon index, showed no difference between Control and TUDCA treatment. Moreover, PCoA result demonstrated that there was no clear separation of bacterial communities structure between the CON and TUDCA groups, which meant no significant differences in microbiota beta diversity between the 2 groups (Fig. 5C). These above results indicated that TUDCA supplementation had no significant effect on diversity of gut microbiota of weaned piglets. In addition, the analysis of relative abundance of bacteria at the phylum level indicated that Firmicutes and Bacteroidetes were the dominant phylums (Fig. 5D). Although TUDCA supplementation did not altered the relative abundance of gut microbiota at the phylum level, it increased the relative abundance of *Parabacteroides* and *Mucispirillum* and decreases the relative abundance of *Streptococcus* and *Treponema 2* at the genus level (*P* < 0.05, Fig. 5E and F).

### Correlations between the differential gut bacteria and serum metabolites

**Fig. 6:**
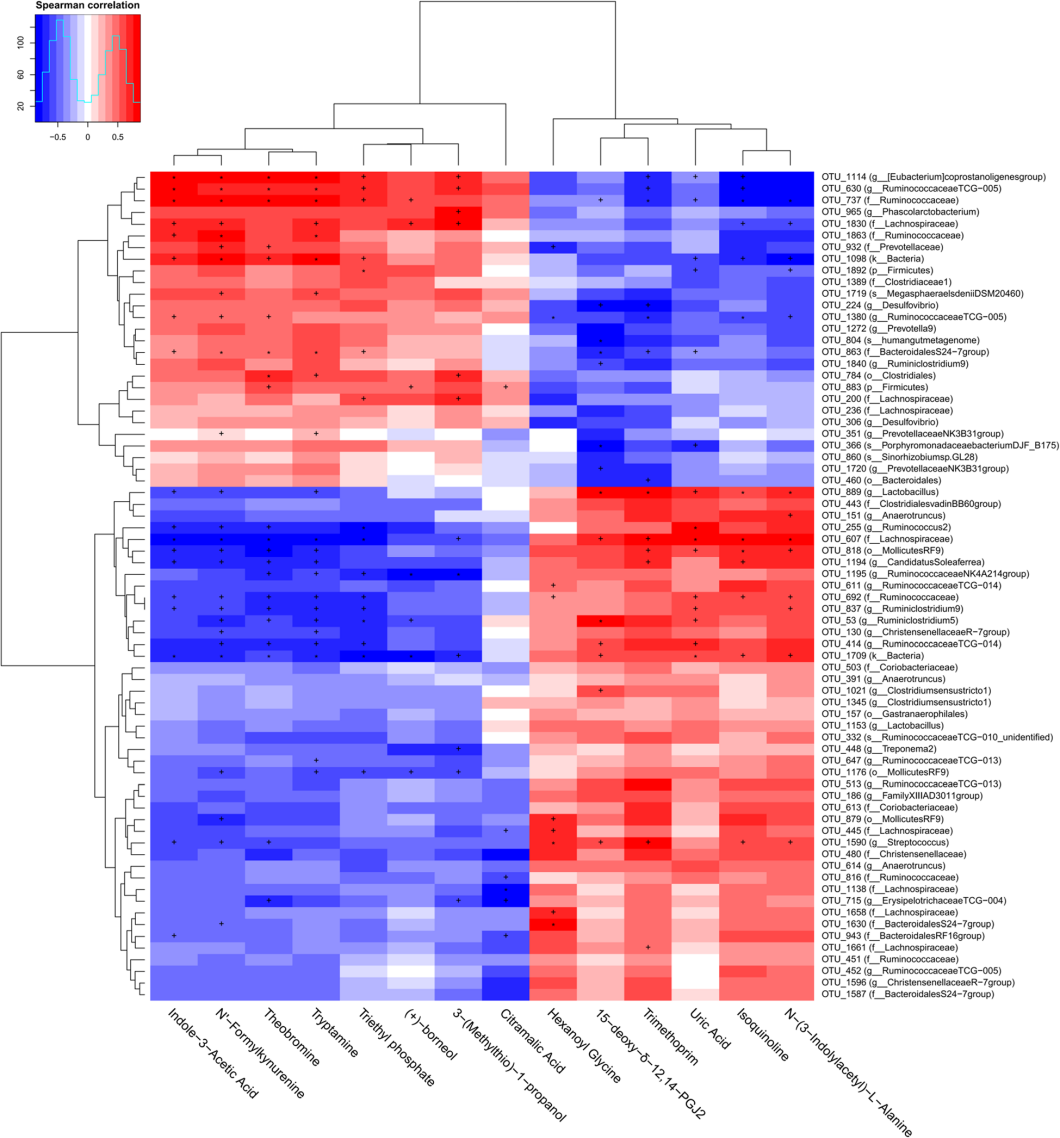
Correlations between serum main differential metabolites and differential operational taxonomic units (OTU) of weaned piglets (*n* = 6). Blue and red cells represent negative and positive correlations, respectively. The significant correlations are indicated by “+” and “*” (+*P* < 0.05 and *P < 0.01)

To explore the relationship between gut microbiota and serum metabolites, Spearman correlation analysis between the serum differential metabolites and the 71 differential OTUs was performed. As shown in Fig. 6, red represented positive correlation and blue represented negative correlation. In the figure, we can find that the color of up-regulated metabolites is clearly distinguished from the color of down-regulated serum metabolites, indicating that the correlation between differential OTUs and up-regulated metabolites was just the opposite to the correlation between the differential OTUs and down-regulated serum metabolites. Moreover, each differential metabolite was significantly correlated with multiple differential OTUs. The above results indicated that these differential gut bacteria were closely related to and might contribute to the alterations of serum metabolic profiles in response to TUDCA treatment.

### TUDCA improved *E. coli*-induced epithelial barrier impairment and enhanced TGR5 expression in IPEC-J2 cells

**Fig. 7:**
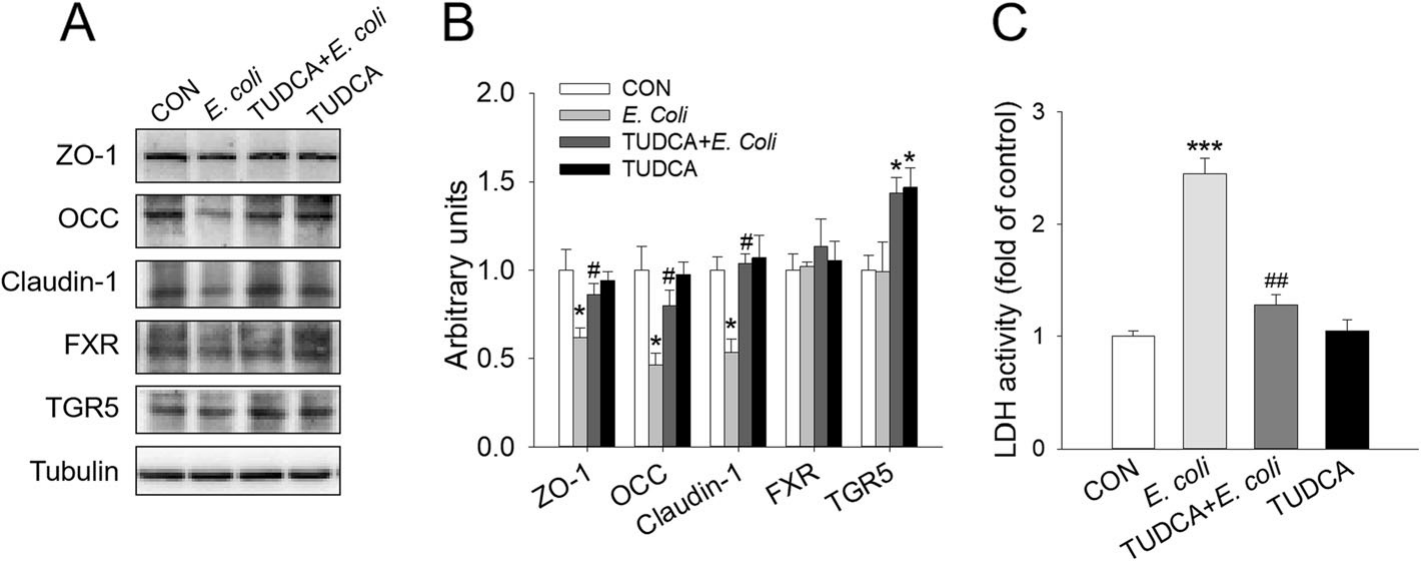
TUDCA improved *E. coli*-induced epithelial barrier impairment and enhanced TGR5 expression in IPEC-J2 cells. **A** Western blot analysis of ZO-1, OCC, Claudin-1, FXR and TGR5 in IPEC-J2 cells. Tubulin was used as the loading control. **B** Mean ± SEM of immunoblotting bands of ZO-1, OCC, Claudin-1, FXR and TGR5 (*n* = 4). **C** LDH activity in the supernatant of IPEC-J2 cells, expressed as fold of control. *P < 0.05 and ***P < 0.001 versus the control (CON) group, #*P* < 0.05 and ##*P* < 0.01versus the *E. coli* group

An epithelial barrier impairment model of IPEC-J2 cells induced by *E. coli* was used to study the mechanism of TUDCA in protecting intestinal barrier function at the cellular level. The IPEC-J2 cells were treated with 100 μmol/L TUDCA according to the pre-test results. As shown in Fig. 7A and B, *E. coli* significantly reduced the expression of TJ proteins, such as ZO-1, Claudin-1 and OCC in IPEC-J2 cells, while 100 μmol/L TUDCA significantly reversed the decrease in TJ proteins expression induced by *E. coli.* Meanwhile, TUDCA also alleviated *E. coli-*induced increase in LDH activity in cell supernatant (Fig. 7C), implying that TUDCA attenuated the *E. coli*-induced increase in cell permeability. In addition, 100 μmol/L TUDCA could significantly increase the protein expression of TGR5 in IPEC-J2 cells, with no significant effect on that of FXR (Fig. 7A and B). The above results implied that the effect of TUDCA on improving epithelial barrier impairment of IPEC-J2 cells induced by *E. coli* might be related to the activation of the bile acid receptor TGR5.

### Knockdown of TGR5 abolished the TUDCA-improved epithelial barrier impairment in *E. coli*-treated IPEC-J2 cells

**Fig. 8:**
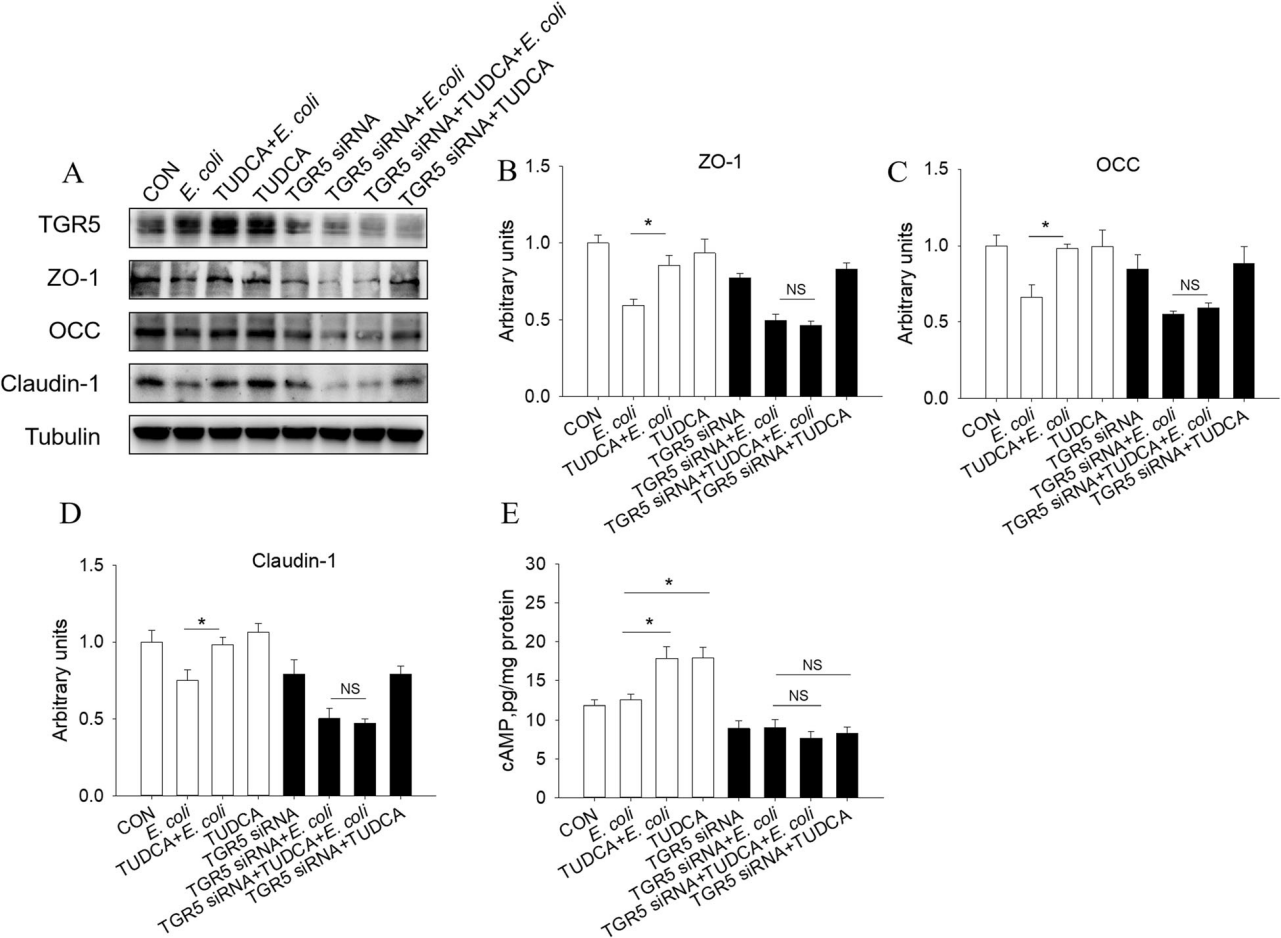
Knockdown of TGR5 abolished the TUDCA-improved epithelial barrier impairment in *E. coli*-treated IPEC-J2 cells. **A**-**D** Western blotting measurements of the protein levels of ZO-1, OCC and Claudin-1 in IPEC-J2 cells after treatment with TUDCA and/or *E. coli* and/or TGR5 siRNA. Tubulin was used as the loading control (*n* = 4). **E** The intracellular cAMP level in IPEC-J2 cells (*n* = 6). ∗*P* < 0.05. NS = not significant

To further confirm whether TGR5 was involved in TUDCA-improved epithelial barrier impairment induced by *E. coli* in IPEC-J2 cells, the specific TGR5 siRNA was used to knock down the expression of TGR5 in IPEC-J2 cells. As shown in Fig. 8A and B, the protein expression of TGR5 decreased significantly after TGR5 siRNA interference. The results showed that after interference with TGR5, TUDCA failed to improve the *E. coli*-induced decrease of protein expression of ZO-1 OCC and Claudin-1 in IPEC-J2 cells (Fig. 8A-D). In agreement, the TUDCA-induced increase in the intracellular cAMP concentration was blocked in the presence of TGR5 siRNA (Fig. 8E). These results provided evidence that TGR5 was responsible for the TUDCA-improved epithelial barrier impairment of IPEC-J2 cells induced by *E. coli.*

### TGR5-MLCK signaling pathway was involved in the TUDCA-improved epithelial barrier impairment in *E. coli*-treated IPEC-J2 cells

**Fig. 9:**
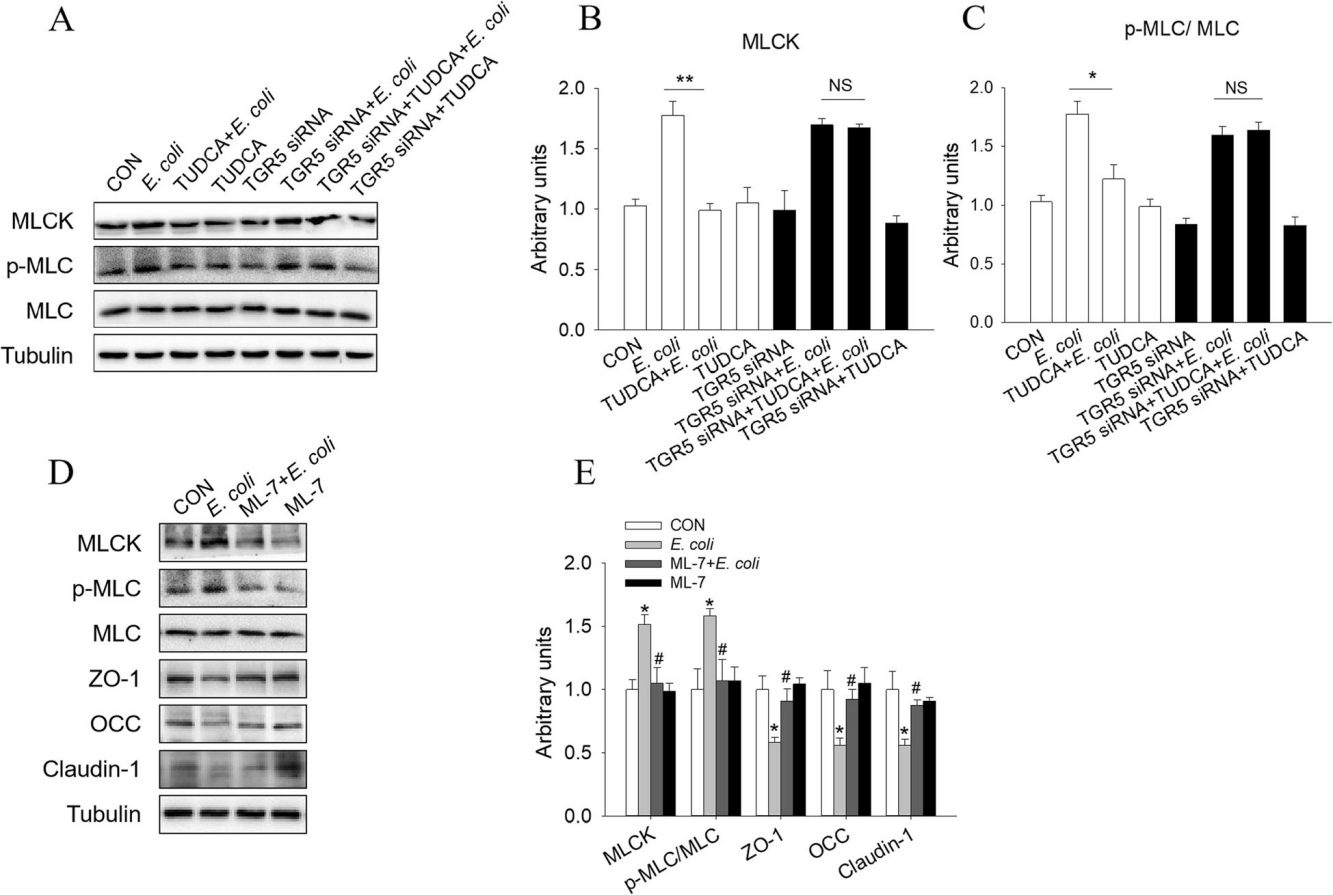
TGR5-MLCK signaling pathway was involved in TUDCA-improved epithelial barrier impairment in *E. coli*-treated IPEC-J2 cells. **A**-**C** Western blotting measurements of the protein levels of MLCK, p-MLC and MLC in IPEC-J2 cells after treatment with TUDCA and/or *E. coli* and/or TGR5 siRNA. Tubulin was used as the loading control (*n* = 4) ∗*P* < 0.05 and ∗∗*P* < 0.01. NS = not significant. **D** and **E** Western blotting measurements of the protein levels of MLCK, p-MLC/MLC, ZO-1, OCC and Claudin-1 in the presence of *E. coli* and/or ML-7. Tubulin was used as the loading control (*n* = 4). ∗*P* < 0.05 versus the control group. #*P* < 0.05 versus the *E. coli* group

To further determine whether the MLCK signaling pathway is involved in TUDCA-improved epithelial barrier impairment induced by *E. coli* in IPEC-J2 cells, as well as its relation with TGR5, we examined activation of the MLCK signaling pathway in the presence of TUDCA and/or *E. coli* and/or TGR5 siRNA. As shown in the Fig. 9A-C, the MLCK signaling pathway was activated by *E. coli*, with increased MLCK expression and an elevated p-MLC/MLC ratio. However, the *E. coli*-induced the MLCK signaling pathway activation was abolished by TUDCA treatment. After interference with TGR5, TUDCA was no longer able to suppress the *E. coli*-induced activation of the MLCK signaling pathway, with no difference in MLCK expression and p-MLC/MLC ratio between *E. coli* and *E. coli* + TUDCA groups. These results indicated that the effect of TUDCA on blocking *E. coli*-induced the activation of MLCK signaling pathway was TGR5-dependent.

In addition, inhibition of MLCK with the inhibitor ML-7 significantly blocked the *E. coli*-activated MLCK signaling pathway, which was similar to the effects of TUDCA (Fig. 9D, E). Furthermore, ML-7 also reversed the decreased expression of ZO-1, OCC and claudin-1 induced by *E. coli*. These results revealed that TUDCA improved *E. coli*-induced impairment of IPEC-J2 cell barrier function through the TGR5-MLCK signaling pathway.

## Discussion

In this study, we provided evidence that TUDCA improved intestinal barrier function associated with TGR5-MLCK pathway and the alteration of serum metabolites and gut microbiota in weaned piglets. Although having no significant effect on the growth performance of weaned piglets (such as the final BW, ADG, ADFI and F/G), TUDCA supplementation significantly reduced the diarrhea incidence of weaned piglets. To investigate the underlying mechanism by which TUDCA alleviates post-weaning diarrhea, we determined the effect of TUDCA on gut barrier integrity and function. Studies have shown that weaning stress could cause intestinal damage, which is manifested as destroyed the intestinal morphology, increase in intestinal permeability [28]. Our study showed that TUDCA supplementation improved the intestinal morphology, increased the secretion of mucopolysaccharides and expressions of tight junction proteins and reduce intestinal permeability. The intestinal tight junctions, include occludin, claudins, and scaffolding proteins like zona occludens (ZO), are the most important structure of the epithelial barrier and play an important role in maintaining the stability of intestinal epithelial barrier and permeability [29]. Intestinal mucosal permeability indicators, including DAO and LPS, are the main basis to evaluate the intestinal barrier function [30, 31]. Collectively, our results indicated that TUDCA supplementation contributed to the reconstruction of intestinal morphology and enhanced intestinal barrier function and thereby reduced the diarrhea incidence of of weaned piglets.

Inflammatory cytokines not only play a crucial role in inflammatory and immune responses, but also regulate the integrity of the intestinal barrier, which is closely related to the occurrence of diarrhea [32]. In the current study, our results showed that TUDCA supplementation reduced intestinal inflammation and promoted immunity function in weaned piglets. Consistent with our findings, it was also reported that TUDCA had anti-inflammatory effects and could alleviate increasing inflammatory cytokines expression in DSS-induced colitis mice [17]. Some researchers have studied the anti-inflammatory mechanism of TUDCA and found that TUDCA alleviates acute and chronic colon inflammation by reducing ER stress [18, 19]. Another study has shown that TUDCA induces cellular apoptosis by inhibition of NF-κB-regulated anti-apoptotic genes [33]. In addition, our study also found that the protein expression of TGR5 in the intestine was elevated. It was reported that TGR5 is a mediator of the immunosuppressive effects of bile acids and plays an anti-inflammatory effect by inhibiting the proinflammatory NF-κB and cytokines production by macrophages [34, 35]. Our findings suggested that TUDCA may exert an anti-inflammatory effect through TGR5, but its specific mechanism remains to be further studied.

The OPLS-DA scores plot results showed that the serum metabolic profile of weaned piglets in the TUDCA group was significantly different from that in the CON group. Among the 14 serum differential metabolites identified, we noticed two upregulated serum differential metabolites after TUDCA supplementation, indole-3-acetic acid and N′-formylkynurenine, are microbiota-derived tryptophan metabolites. Interestingly, KEGG pathway analysis also showed that TUDCA supplementation affected tryptophan metabolism of weaned piglets. Tryptophan and bile acids are two important gut microbial metabolites. It has been found that certain gut bacteria, such as *Bififidobacterium* and *Blautia* species, are closely associated with both tryptophan and bile acids metabolism [36], so alterations in bile acid metabolic, including bile acid intake, are often accompanied by changes in tryptophan metabolites. Metabolites produced by the intestinal flora metabolizing tryptophan, especially indole derivatives, play a key role in maintaining the integrity of the intestinal barrier and protecting the mucosa from inflammation, which is beneficial to intestinal health [37–39]. In addition, theobromine has been reported to inhibit the growth of harmful bacteria in rats, including *Escherichia coli*, *Streptococcus* spp., and *Clostridium histolyticum-C* [40]. These results indicated that TUDCA-improved intestinal barrier function of weaned piglets might be mediated, at least in part, by alteration of serum metabolic profiles.

The composition of intestinal flora is also an important factor affecting intestinal barrier function [41]. In the present study, we found that *Parabacteroides* and *Mucispirillum* at the genus level were markedly increased in response to TUDCA. *Parabacteroides* was reported to be negatively correlated with body weight gain and hepatic triglyceride content [42]. The increase relative abundance of *Parabacteroides* might be one of the reasons why TUDCA supplementation did not improve the growth performance of weaned piglets. *Mucispirillum* has been observed to be positively correlated with intestinal proliferation and differentiation [43]. Moreover, *Mucispirillum* is closely related to the production and secretion of T cell-dependent IgA, which is the main component of mucosal immune barrier and mediate intestinal mucosal immunity [44]. Therefore, the increased relative abundance of *Mucispirillum* indicated the enhancement of intestinal proliferation, differentiation and mucosal immunity, and the decrease of diarrhea rate and the enhancement of intestinal barrier functions of weaned piglets in the TUDCA group might be related to the increased relative abundance of *Mucispirillum* in the intestine. In addition, TUDCA supplementation reduced the relative abundance of harmful intestinal bacteria, *Streptococcus* and *Treponema 2* at the genus level*. Streptococcus* is a common and harmful bacterium that can cause meningitis, septicaemia, pneumonia and arthritis in pigs, causing significant economic losses to the pig industry [45]. Therefore, changes in the relative abundance of these intestinal bacteria of weaned piglets might be closely related to the improvement of intestinal barrier function after TUDCA supplementation.

A correlation analysis between serum differential metabolites and intestinal differential OTUs was carried out. Our study found that the correlation between the increased serum differential metabolites and the differential OTUs was just opposite to the correlation between the decreased serum differential metabolites and the differential OTUs. Moreover, most of the serum differential OTUs were positively or negatively correlated with each serum differential metabolites, and many of them were significantly correlated, indicating that the changes in certain intestinal bacteria was contribute to the alteration of serum metabolites.

The results of the weaned piglet feeding experiment showed that TUDCA improved intestinal barrier function and reduced the relative abundance of intestinal harmful bacteria of weaned piglets. To further investigate the underlying mechanism of TUDCA improving the intestinal barrier function of weaned piglets, *E. coli-*induced IPEC-J2 cells were used as a model system. Our results showed that *E. coli-*induced intestinal barrier function impairment in IPEC-J2 cells was abolished by TUDCA pretreatment. Numerous studies have shown that TUDCA is an agonist of TGR5 and can act by activating TGR5 and its downstream signaling pathways [46, 47]. Consistent with these studies, we found that TUDCA treatment increased TGR5 expression in IPEC-J2 cells. Then the TGR5-knockdown experiment was conducted for further verify that TGR5 was involved in the process of TUDCA improving *E. coli-*induced intestinal barrier function impairment in IPEC-J2 cells. In line with our result, a previous study has reported that TGR5 is involved in regulating the integrity of intestinal barrier and its absence manifests by an increased intestinal permeability,while its activation attenuates colon inflammation in rodent models of colitis [48].

It has been reported that epithelial MLCK signaling pathway is crucial to tight junction barrier regulation [49, 50]. MLCK has been demonstrated to be closely associated with the LPS-induced impairment of the intestinal epithelial barrier [21, 51]. Similarly, in our present study, we observed that the MLCK pathway was involved in the *E. coli*-induced impairment of intestinal epithelial barrier. However, the *E. coli*-induced activation of the MLCK signaling pathway was abolished by TUDCA, presenting similar effects to that of MLCK inhibitor, ML-7. In addition, our findings showed that TUDCA blocked *E. coli*-induced activation of the MLCK signaling pathway in a TGR5-dependent manner. Collectively, we concluded that TUDCA alleviated *E. coli*-induced impairment of the intestinal epithelial barrier in IPEC-J2 cells through the TGR5-MLCK signaling pathway.

## Conclusions

In conclusion, these findings showed that TUDCA improved intestinal barrier function associated with TGR5-MLCK pathway and the alteration of serum metabolites and gut bacteria in weaned piglets. These data provided new evidence into the regulation of bile acids on the intestinal barrier function and suggested the potential application of TUDCA in improving intestinal health in piglet production.

## Data Availability

All data generated or analyzed during this article are available from the corresponding author upon reasonable request.

## Abbreviations

ADFI: Average daily feed intake
ADG: Average daily gain
BAs: Bile acids
BW: Body weight
CA: Cholic acid
CDCA: Chenodeoxycholic acid
CFU: Colony-forming units
CON: Control
DAO: Diamine oxidase
DCA: Deoxycholic acid
DMSO: Dimethyl sulfoxide
ER: Endoplasmic reticulum
FDA: Food and Drug Administration
F/G: Feed/gain ratio
FXR: Farnesoid X receptor
HDCA: Hyodeoxycholic acid
H&E: Haematoxylin and eosin
KEGG: Kyoto Encyclopedia of Genes and Genomes
LB: Luria-Bertani
LCA: Lithocholic acid
LC-MS/MS: Liquid chromatography tandem mass spectrometry
LDH: Lactate dehydrogenase
LPS: Lipopolysaccharide
MLCK: Myosin light chain kinase
OCC: Occludin
OPLS-DA: Orthogonal partial least squares-discriminant analysis
OTU: Operational taxonomic unit
PBS: Phosphate buffer solution
PAS: Periodic acid-schiff
PCoA: Principal component analysis
SEM: Standard error of the mean
sIgA: Secretory immunoglobulin A
TGR5: Takeda G-coupled protein receptor 5
TJ: Tight junction
TUDCA: Tauroursodeoxycholic acid
UDCA: Ursodeoxycholic acid

## References

[1] Lalles JP, Bosi P, Smidt H, Stokes CR (2007). Nutritional management of gut health in pigs around weaning. Proc Nutr Soc.

[2] Gresse R, Chaucheyras-Durand F, Fleury MA, Tom VDW, Forano E, Blanquet-Diot S (2017). Gut microbiota dysbiosis in postweaning piglets: understanding the keys to health. Trends Microbiol.

[3] Schneider KM, Albers S, Trautwein C (2018). Role of bile acids in the gut-liver axis. J Hepatol.

[4] Ahmad TR, Haeusler RA (2019). Bile acids in glucose metabolism and insulin signalling-mechanisms and research needs. Nat Rev Endocrinol.

[5] Li W, Hang SY, Fang Y, Bae S, Zhang YC, Zhang MH, Wang G, McCurry MD, Bae M, Paik D, Franzosa EA, Rastinejad F, Huttenhower C, Yao L, Devlin AS, Huh JR (2021). A bacterial bile acid metabolite modulates Treg activity through the nuclear hormone receptor NR4A1. Cell Host Microbe.

[6] Xiang JW, Zhang ZY, Xie HY, Zhang CC, Bai Y, Cao H, Che Q, Guo J, Su Z (2021). Effect of different bile acids on the intestine through enterohepatic circulation based on FXR. Gut Microbes.

[7] Winston JA, Theriot CM (2020). Diversification of host bile acids by members of the gut microbiota. Gut Microbes.

[8] Lucas LN, Barrett K, Kerby RL, Zhang Q, Cattaneo LE, Stevenson D (2021). Dominant bacterial phyla from the human gut show widespread ability to transform and conjugate bile acids. mSystems.

[9] Wahlstrom A, Sayin SI, Marschall HU, Backhed F (2016). Intestinal crosstalk between bile acids and microbiota and its impact on host metabolism. Cell Metab.

[10] Zheng XJ, Zhou KJ, Zhang YJ, Han XL, Zhao AH, Liu JJ, Qu C, Ge K, Huang F, Hernandez B, Yu H, Panee J, Chen T, Jia W, Jia W (2018). Food withdrawal alters the gut microbiota and metabolome in mice. FASEB J.

[11] Bahar R, Wong KA, Liu CH, Bowlus CL (2018). Update on new drugs and those in development for the treatment of primary biliary cholangitis. Gastroenterol Hepatol.

[12] Daruich A, Picard E, Boatright JH, Behar-Cohen F (2019). Review: the bile acids urso- and tauroursodeoxycholic acid as neuroprotective therapies in retinal disease. Mol Vis.

[13] Lepercq P, Gerard P, Beguet F, Raibaud P, Grill JP, Relano P (2004). Epimerization of chenodeoxycholic acid to ursodeoxycholic acid by clostridium baratii isolated from human feces. FEMS Microbiol Lett.

[14] Kusaczuk M (2019). Tauroursodeoxycholate-bile acid with chaperoning activity: molecular and cellular effects and therapeutic perspectives. Cells..

[15] Ozcan U, Cao Q, Yilmaz E, Lee AH, Iwakoshi NN, Ozdelen E (2004). Endoplasmic reticulum stress links obesity, insulin action, and type 2 diabetes. Science..

[16] Zangerolamo L, Vettorazzi JF, Rosa LRO, Carneiro EM, Barbosa HCL (2021). The bile acid TUDCA and neurodegenerative disorders: an overview. Life Sci.

[17] Lien VDB, Hindryckx P, Devisscher L, Devriese S, Van Welden S, Holvoet T (2017). Ursodeoxycholic acid and its taurine- or glycine-conjugated species reduce colitogenic dysbiosis and equally suppress experimental colitis in mice. Appl Environ Microbiol.

[18] Cao SS, Zimmermann EM, Chuang BM, Song B (2013). The unfolded protein response and chemical chaperones reduce protein misfolding and colitis in mice. Gastroenterology..

[19] Laukens D, Devisscher L, Bossche LV, Hindryckx P, Vandenbroucke RE, Vandewynckel YP (2014). Tauroursodeoxycholic acid inhibits experimental colitis by preventing early intestinal epithelial cell death. Lab Investig.

[20] National Research Council (NRC) (2012). Nutrient requirements of swine: 11th revised edition.

[21] Song M, Ye JY, Zhang FL, Su H, Yang XH, He HW, Liu F, Zhu X, Wang L, Gao P, Shu G, Jiang Q, Wang S (2019). Chenodeoxycholic acid (CDCA) protects against the lipopolysaccharide-induced impairment of the intestinal epithelial barrier function via the FXR-MLCK pathway. J Agric Food Chem.

[22] Feng WQ, Wu YC, Chen GX, Fu SP, Li B, Huang BX, Wang D, Wang W, Liu J (2018). Sodium butyrate attenuates diarrhea in weaned piglets and promotes tight junction protein expression in colon in a GPR109A-dependent manner. Cell Physiol Biochem.

[23] Wang SB, Liang XW, Yang QY, Fu X, Zhu MJ, Rodgers BD, Jiang Q, Dodson MV, du M (2017). Resveratrol enhances brown adipocyte formation and function by activating AMP-activated protein kinase (AMPK) alpha1 in mice fed high-fat diet. Mol Nutr Food Res.

[24] Deng QQ, Shao YR, Wang QY, Li JZ, Li YL, Ding XQ (2020). Effects and interaction of dietary electrolyte balance and citric acid on the intestinal function of weaned piglets. J Anim Sci.

[25] Meng YY, Zhang J, Zhang FL, Ai W, Zhu XT, Shu G, Wang L, Gao P, Xi Q, Zhang Y, Liang X, Jiang Q, Wang S (2017). Lauric acid stimulates mammary gland development of pubertal mice through activation of GPR84 and PI3K/Akt signaling pathway. J Agric Food Chem.

[26] Sun LY, Kang Q, Pan YM, Li N, Wang X, He YQ, Wang H, Yu D, Xie H, Yang L, Lu Y, Jin P, Sheng J (2019). Serum metabolite profiling of familial adenomatous polyposis using ultra performance liquid chromatography and tandem mass spectrometry. Cancer Biol Ther.

[27] Zheng P, Zeng B, Zhou C, Liu M, Fang Z, Xu X, Zeng L, Chen J, Fan S, du X, Zhang X, Yang D, Yang Y, Meng H, Li W, Melgiri ND, Licinio J, Wei H, Xie P (2016). Gut microbiome remodeling induces depressive-like behaviors through a pathway mediated by the host's metabolism. Mol Psychiatry.

[28] Smith F, Clark JE, Overman BL, Tozel CC, Huang JH, Rivier JE (2010). Early weaning stress impairs development of mucosal barrier function in the porcine intestine. Am J Physiol Gastrointest Liver Physiol.

[29] Paradis T, Bègue H, Basmaciyan L, Dalle F, Bon F (2021). Tight junctions as a key for pathogens invasion in intestinal epithelial cells. Int J Mol Sci.

[30] Qin LS, Ji W, Wang JL, Li B, Hu JP, Wu X (2019). Effects of dietary supplementation with yeast glycoprotein on growth performance, intestinal mucosal morphology, immune response and colonic microbiota in weaned piglets. Food Funct.

[31] Wu MM, Xiao H, Ren WK, Yin J, Tan B, Liu G, Li L, Nyachoti CM, Xiong X, Wu G (2014). Therapeutic effects of glutamic acid in piglets challenged with deoxynivalenol. PLoS One.

[32] Al-Sadi R, Boivin M, Ma T (2009). Mechanism of cytokine modulation of epithelial tight junction barrier. Front Biosci (Landmark Ed).

[33] Kim YH, Kim JH, Kim BG, Lee KL, Kim JW, Koh SJ (2019). Tauroursodeoxycholic acid attenuates colitis-associated colon cancer by inhibiting nuclear factor kappaB signaling. J Gastroenterol Hepatol.

[34] Keitel V, Donner M, Winandy S, Kubitz R, Haussinger D (2008). Expression and function of the bile acid receptor TGR5 in Kupffer cells. Biochem Biophys Res Commun.

[35] Pols TW, Nomura M, Harach T, Lo Sasso G, Oosterveer MH, Thomas C (2011). TGR5 activation inhibits atherosclerosis by reducing macrophage inflammation and lipid loading. Cell Metab.

[36] Golubeva AV, Joyce SA, Moloney G, Burokas A, Sherwin E, Arboleya S, Flynn I, Khochanskiy D, Moya-Pérez A, Peterson V, Rea K, Murphy K, Makarova O, Buravkov S, Hyland NP, Stanton C, Clarke G, Gahan CGM, Dinan TG, Cryan JF (2017). Microbiota-related changes in bile acid & tryptophan metabolism are associated with gastrointestinal dysfunction in a mouse model of autism. EBioMedicine..

[37] Postler TS, Ghosh S (2017). Understanding the holobiont: how microbial metabolites affect human health and shape the immune system. Cell Metab.

[38] Marsland BJ (2016). Regulating inflammation with microbial metabolites. Nat Med.

[39] Krishnan S, Ding Y, Saedi N, Choi M, Sridharan GV, Sherr DH, Yarmush ML, Alaniz RC, Jayaraman A, Lee K (2018). Gut microbiota-derived tryptophan metabolites modulate inflammatory response in hepatocytes and macrophages. Cell Rep.

[40] Martín-Peláez S, Camps-Bossacoma M, Massot-Cladera M, Rigo-Adrover M, Franch À, Pérez-Cano FJ, Castell M (2017). Effect of cocoa’s theobromine on intestinal microbiota of rats. Mol Nutr Food Res.

[41] Guzior DV, Quinn RA (2021). Review: microbial transformations of human bile acids. Microbiome..

[42] Carbajo-Pescador S, Porras D, Garcia-Mediavilla MV, Martinez-Florez S, Juarez-Fernandez M, Cuevas MJ (2019). Beneficial effects of exercise on gut microbiota functionality and barrier integrity, and gut-liver crosstalk in an in vivo model of early obesity and non-alcoholic fatty liver disease. Dis Model Mech.

[43] Jin G, Tang Q, Ma JH, Liu X, Zhou BQ, Sun Y (2021). Maternal emulsifier P80 intake induces gut dysbiosis in offspring and increases their susceptibility to colitis in adulthood. mSystems.

[44] Bunker JJ, Flynn TM, Koval JC, Shaw DG, Meisel M, McDonald BD (2015). Innate and adaptive humoral responses coat distinct commensal bacteria with immunoglobulin A. Immunity..

[45] Dinesh M, Thakor J, Vishwa KV, Pathak M, Patel SK, Kumar P, Qureshi S, Singh KP, Sahoo M (2020). Pathology and diagnosis of Streptococcus suis infections in pre-weaned piglets. Indian J Vet Pathol.

[46] Vettorazzi JF, Ribeiro RA, Borck PC, Branco RCS, Soriano S, Merino B, Boschero AC, Nadal A, Quesada I, Carneiro EM (2016). The bile acid TUDCA increases glucose-induced insulin secretion via the cAMP/PKA pathway in pancreatic beta cells. Metabolism..

[47] Yanguas-Casás N, Barreda-Manso MA, Nieto-Sampedro M, Romero-Ramírez L (2016). TUDCA: an agonist of the bile acid receptor GPBAR1/TGR5 with anti-inflammatory effects in microglial cells. J Cell Physiol.

[48] Cipriani S, Mencarelli A, Chini MG, Distrutti E, Renga B, Bifulco G, Baldelli F, Donini A, Fiorucci S (2011). The bile acid receptor GPBAR-1 (TGR5) modulates integrity of intestinal barrier and immune response to experimental colitis. PLoS One.

[49] Chelakkot C, Ghim J, Ryu SH (2018). Mechanisms regulating intestinal barrier integrity and its pathological implications. Exp Mol Med.

[50] Buckley A, Turner JR (2018). Cell biology of tight junction barrier regulation and mucosal disease. Cold Spring Harb Perspect Biol.

[51] Nighot M, Al-Sadi R, Guo S, Rawat M, Nighot P, Watterson MD (2017). Lipopolysaccharide-induced increase in intestinal epithelial tight permeability is mediated by toll-like receptor 4/myeloid differentiation primary response 88 (MyD88) activation of myosin light chain kinase expression. Am J Pathol.

